# Short-term Psychodynamic Psychotherapy in Addition to Standard Medical Therapy Increases Clinical Remission in Adolescents and Young Adults with Inflammatory Bowel Disease: a Randomised Controlled Trial

**DOI:** 10.1093/ecco-jcc/jjad145

**Published:** 2023-08-24

**Authors:** Francesco Milo, Chiara Imondi, Carmen D’Amore, Giulia Angelino, Daniela Knafelz, Fiammetta Bracci, Luigi Dall’Oglio, Paola De Angelis, Paola Tabarini

**Affiliations:** Clinical Psychology Unit, Bambino Gesù Children’s Hospital, IRCCS, Rome, Italy; Digestive Endoscopy Unit, Bambino Gesù Children’s Hospital, IRCCS, Rome, Italy; Clinical Epidemiology Unit, Bambino Gesù Children’s Hospital, IRCCS, Rome, Italy; Digestive Endoscopy Unit, Bambino Gesù Children’s Hospital, IRCCS, Rome, Italy; Hepatology, Gastroenterology and Nutrition Unit, Bambino Gesù Children’s Hospital, IRCCS, Rome, Italy; Hepatology, Gastroenterology and Nutrition Unit, Bambino Gesù Children’s Hospital, IRCCS, Rome, Italy; Digestive Surgery Unit, Bambino Gesù Children’s Hospital, IRCCS, Rome, Italy; Digestive Endoscopy Unit, Bambino Gesù Children’s Hospital, IRCCS, Rome, Italy; Clinical Psychology Unit, Bambino Gesù Children’s Hospital, IRCCS, Rome, Italy

**Keywords:** Psychotherapy, effectiveness, randomised controlled trial, paediatric

## Abstract

**Background:**

Inflammatory bowel diseases [IBD] are chronic and pervasive conditions of the gastrointestinal tract with a rising incidence in paediatric and young adult populations. Evidence suggests that psychological disorders might be associated with relapse of disease activity. This study aims to evaluate the efficacy of short-term psychodynamic psychotherapy [STPP] in addition to standard medical therapy [SMT] in maintaining clinical remission in adolescents and young adults [AYA] with quiescent IBD, compared with SMT alone.

**Methods:**

A two-arm, single-centre, randomised, controlled trial was conducted in 60 IBD AYA in clinical remission. Patients were randomised to receive an 8-week STPP + SMT [*n* = 30] or SMT alone [*n* = 30]. The primary outcome was the steroid-free remission rate at 52 weeks after treatment. Secondary outcomes included the overall hospitalisation rate within 52 weeks after treatment, and medication adherence obtained from patient’s electronic medical records.

**Results:**

Intention-to-treat analysis showed significant improvement in maintaining disease remission rates in the 8-week STPP + SMT group compared with the control one. The proportion of patients maintaining steroid-free remission at 52 weeks was higher in patients in STTP group [93.1%] compared with patients randomised to control group [64.3%; *p* = 0.01]. There were no significant differences in secondary outcomes, except for depression reduction in STPP + SMT group.

**Conclusions:**

An 8-week STPP intervention in addition to SMT effectively increases the steroid-free remission rates in AYA with quiescent IBD. Results do not support effects for other secondary outcomes, except for depression reduction.

## 1. Introduction

Inflammatory bowel disease [IBD] is a chronic inflammatory disorder that includes Crohn’s disease [CD] and ulcerative colitis [UC], two inflammatory conditions of the gastrointestinal tract characterised by periods of active inflammation followed by periods of clinical remission. These disorders are often diagnosed in adolescence and young adulthood, and present intestinal symptoms such as weight loss, abdominal pain, and bloody diarrhoea, or extra-intestinal symptoms such as poor growth, anaemia, or other extra-intestinal manifestations.^1^

Recent studies described a temporal relationship between the presence of anxiety and depression, and the onset of clinical disease activity, in a large cohort of IBD patients.^2^ Other studies have also suggested an association between active disease and the onset of psychological comorbidity over time.^3^ Furthermore, recent observational data suggest a substantial brain-gut axis disorder in IBD, highlighting the need to integrate psychological treatments into a biopsychosocial model of care for IBD to improve the natural history of the disease.^4^

There are several available research studies evaluating the role of short-term psychodynamic psychotherapy [STPP] in addition to standard medical care in inflammatory bowel syndrome.^5,6^ Psychodynamic approach focuses on unconscious processes that influence patient’s behaviour. The goals of psychodynamic therapy are client self-awareness and understanding of the influence of the past on present behaviour. Unlike other forms of psychotherapy, psychodynamic therapy focuses on emotional awareness and defences analysis. The relationship between therapist and patient is used as a window into problematic relationship patterns in patient’s life. Furthermore, STPP is shorter and more focused, and it is delivered face to face.^7^ Several studies suggest that patients who received STPP reported more symptom relief than those who received only medical treatment, and these group differences persisted both at 3-month and 1-year follow-ups. However, there is only one study evaluating STPP as an additional treatment to standard medical care in IBD adult patients, reporting a trend toward fewer surgical procedures and fewer relapses in the intervention group.^8^ The application of STPP in addition to standard medical care showed encouraging results in other chronic health conditions. For instance, individuals with diabetes were able to achieve clinically relevant improvements in glycaemic control through additional STPP, compared with standard care alone.^9^ Furthermore, recent studies demonstrated that STPP improved depressive symptoms and fatigue in patients with breast cancer.^10,11^

The primary purpose of this randomised controlled trial was to examine the efficacy of STPP in addition to standard medical therapy [SMT] to maintain clinical steroid-free remission in adolescents and young adults [AYA] with quiescent IBD, when compared with SMT alone. In addition, we examined improvements in health care use and adherence [ie, secondary outcomes] in patients following STPP, when compared with the controls.

## 2. Patients and Methods

### 2.1. Study design

This is a randomised controlled trial [RCT] of all IBD consecutive adolescents and young adults [aged between 11 and 21 years] followed at Bambino Gesù Children’s Hospital of Rome, who were in clinical remission at the time of recruitment. The study period, from the recruitment to the completion of follow-up, was from September 2021 to December 2022. A paediatric gastroenterologist screened patients for eligibility. Based on this screening procedure, patients with IBD in clinical remission who fulfilled eligibility criteria received an information letter and an informed consent form, as well as a short sociodemographic questionnaire. Those patients who were able to understand and provided written informed consent were included in the study. Patients were randomised into an intervention group receiving STPP in addition to SMT and a control group receiving SMT alone. Assessments in both groups were performed before randomisation [baseline] and directly after 52 weeks after the intervention period [approximately 1 year after baseline]. This study was approved by Bambino Gesù Ethics Committee [protocol no. 2857] and conducted following the principles of the Declaration of Helsinki. The trial was registered on the ISRCTN registry [ISRCTN24678005]. Informed consent was obtained from all the participants and their legal guardians.

### 2.2. Participants

Patients were eligible for inclusion if they had a diagnosis of either CD or UC, as confirmed by endoscopic examination and according to the Porto criteria.^12^ The main selection criterion was the presence of IBD in clinical remission at the time of recruitment. Clinical remission was defined as a Paediatric Crohn’s Disease Activity Index [PCDAI]^13^ score < 10 for patients with CD and as a Paediatric Ulcerative Colitis Activity Index [PUCAI]^14^ score <10 for patients with UC. Eligible patients were aged between 11 and 21 years, similarly distributed according to gender, able to read, write, and speak Italian language, and without expectation of surgery in the upcoming 3 months. Exclusion criteria were severe, cognitive, neurological, and psychiatric co-occurring conditions that could interfere with patients’ participation. Other exclusion criteria were inability to provide informed consent and receiving psychological treatment or psychotropic medication at the time of recruitment [or other psychotropic medication <2 years before recruitment]. The latter was implemented to minimise possible confounding by other psychological/pharmacological treatments.

### 2.3. Intervention

**Figure 1.**
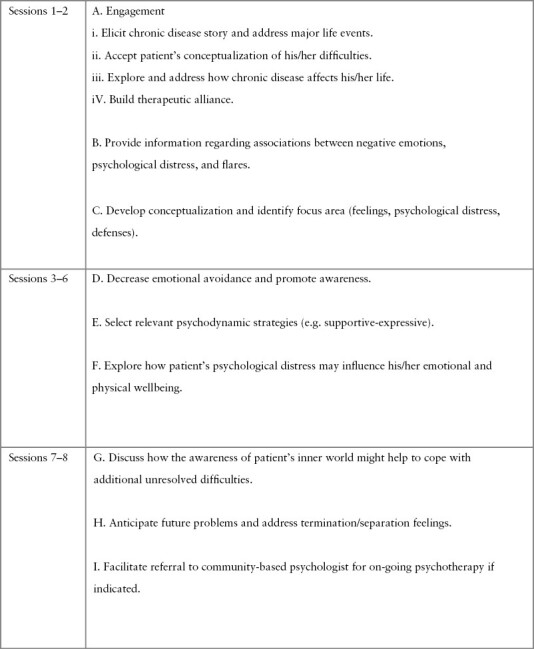
Timeline for completing primary STPP tasks.

Psychological stress can have a detrimental effect on individuals diagnosed with IBD.^15^ Individual with IBD often describe stress as pain trigger.^16^ Indeed, according to a recent study, stress is the most frequent trigger of flares.^17,18^ STPP is a psychotherapy that focuses on troubling feelings or thoughts that interfere with relationships, communication, and/or functioning. Brief [short-term] psychotherapy differs from long-term psychodynamic psychotherapy by being much briefer and time-limited treatment. STPP seeks to increase the patient’s understanding of his or her internal functioning. An STPP therapist would begin by developing a dynamic formulation of the case. This formulation focuses on intrapsychic conflicts and would comprise a specific constellation of dynamic elements: defences, emotional distress, and unconscious feelings, as well as their interrelationships.^19^ Feelings that are perceived as threatening to key relationships [ie, when someone important to us, such as parents, reacts to our emotions by expressing discomfort, withdrawal, or by expressing anger] will evoke emotional distress [ie, anxiety] and hence [that feelings] would be suppressed and/or distorted [defences] in order to maintain the relationship.^20^ Central to the case of a patient with IBD is that physical symptoms [eg, the worry about incontinence in patients with diarrhoea, the fear associated with experiencing physical symptoms] may increase negative emotions leading to increased psychological distress. Our adapted STPP investigates the effects of patient’s personal history and disease on his/her mental health, and vice versa. Compared with other manualised therapeutic approaches, our STPP is personalised: every suffering is different and every intervention is tailored to the patient. Recent literature has identified a bidirectional relationship between negative emotions such as anxiety and sadness with inflammation, emphasising the influence of negative emotions and their contribution to heightened inflammation.^21^ Treatment approach depends if the primary focus is/are: 1] feelings [the therapist explores emotions]; 2] psychological distress, such as anxiety or sadness [the therapist helps regulate emotional distress, and then explore feelings]; and 3] defences [the therapist helps the individual see and let go of defences, then explore feelings that might be behind the defences]. The intervention was developed specifically for this project [by the first and last author] and was based on psychodynamic principles and adapted for the psychosocial needs of individuals with IBD. To ensure the trustworthiness of the intervention, two psychodynamic psychotherapists with advanced postgraduate training [the first and last author] performed all interventions. Weekly briefing sessions have been implemented to disclose difficulties perceived by the patients/therapists or define additional treatment adaptations. Intervention description is summarised in Figure 1.

### 2.4. Control condition

SMT consisted in the continuation of the current medical therapy and regular medical consultations of 15–30 min with the paediatric gastroenterologist every 3 months, in which overall wellbeing, disease activity, and future diagnostic/treatment plans were discussed.

### 2.5. Measures

During baseline assessment, all patients completed a sociodemographic questionnaire, regarding sociodemographic and disease-specific information. All outcomes were assessed at baseline and post intervention [52 weeks after the baseline assessment] times. A paediatric gastroenterologist [CI], who is blinded about participants’ allocation, performed primary and secondary outcomes assessment reviewing patient’s electronic health records. All analyses concerning secondary outcomes were exploratory.

#### 2.5.1. Primary outcome measure

##### 2.5.1.1. Remission maintenance

Remission maintenance was measured using the proportion of participants with steroid-free remission at Week 52 [1 year] between the two treatment groups. Disease activity was measured according to the PCDAI score for patients with CD and the PUCAI score for those with UC. Active disease was defined as a score ≥10 on PCDAI/ PUCAI scores and in addition one or more of the following conditions: the presence of elevated inflammatory markers levels [eg, C-reactive protein levels ≥8 mg/L and faecal calprotectin level ≥100 µg/g] or endoscopic inflammatory findings. A paediatric gastroenterologist, who was blinded to participant assignment, performed disease activity assessment by reviewing patient electronic health records and identifying patients who experienced at least one exacerbation during the 52 weeks following baseline. Disease recurrence was defined as the time interval between baseline and the first flare.

#### 2.5.2. Secondary outcome measure

##### 2.5.2.1. Health care use

To assess health care usage between the two groups, the frequency of hospitalisations over the next 52 weeks after baseline was used.

##### 2.5.2.2. Adherence to medication

Participants were labelled ‘adherent’ if they followed all prescribed medication in the period of 52 weeks after baseline; otherwise, they were labelled ‘non-adherent’. The difference in adherence was evaluated by comparing the proportion of ‘adherent’ and ‘non-adherent’ patients between the two groups.

### 2.6. Psychological measures

#### 2.6.1. Generalised Anxiety Disorder [GAD-7]

The GAD-7 is a seven-item instrument that is used to measure or assess the severity of anxiety symptoms.^15^ Items were scored on a four-point scale [0 = not at all, 1 = several days, 2 = more than half the days, and 3 = nearly every day], with total scores ranging from zero to 21. The recommended screening cutoff was ≥10, corresponding to at least a moderate level of anxiety.^22^

#### 2.6.2. Patient Health Questionnaire [PHQ-9]

The PHQ-9 is a depression symptoms scale consisting of nine questions, and it can be used as a tool for monitoring the depressive symptoms. Items were scored on a four-point scale [0 = not at all, 1 = several days, 2 = more than half the days, and 3 = nearly every day], with total scores ranging from zero to 27. A score of 10 or higher had a sensitivity of 88% and a specificity of 88% for detecting clinically meaningful depression symptoms.^23^

### 2.7. Randomisation

Participants were randomly assigned to an 8-week STPP + SMT or SMT control condition. A data analyst not actively involved in the study design, and blinded concerning characteristics of the study, generated a random allocation sequence and assigned participants to one of the two conditions [1:1 ratio].

### 2.8. Sample size

We hypothesised that STPP + SMT would be superior to SMT alone. Recent data have shown variable rates of remission in paediatric IBD patients receiving standard medical therapy.^24^ Based on results from previous studies on the effectiveness of other forms of psychological therapy in IBD,^25^ we estimated that 75% of the participants in the STPP + SMT group would reach the primary endpoint compared with 40% of the participants in the SMT group at Week 52. A chi square test [or Fisher’s exact test, as appropriate] with a two-sided α of 0.05 will have 80% power to detect the expected difference between treatment arms when the sample size in each group is 30 in each arm.

### 2.9. Statistical analysis

Baseline and outcome variables were summarised using means [M] and standard deviations [SD] for continuous variables and counts and percentages for categorical variables. All missing data were excluded from the analysis. Student’s t test or Pearson’s chi square test was used to compare baseline characteristics between the groups. The primary analysis concerned the comparison of the proportion of participants with steroid-free remission at Week 52 between the two treatment groups [STPP + SMT vs SMT], using Pearson’s chi square test or Fisher’s exact test, as appropriate. All secondary analyses were exploratory. The proportions of hospitalisation and participants who had been defined as ‘adherent’ vs ‘not adherent’ between the two groups, were compared using Fisher’s exact test or chi square test, as appropriate. Paired t test was used to investigate pre-post changes in depressive and anxiety symptoms in the two groups. The effects of the intervention over time were analysed using a two-way analysis of variance [ANOVA], with time as the within-participant factor and treatment as the between-participants factor. A log rank test [Kaplan–Meier curve] was run to determine the remission distribution in STPP + SMT versus SMT alone. Multivariate regression analysed the relationship between treatment allocation and remission maintenance at 1-year follow-up, including variables with statistical significance at baseline between groups. Significance was set at a two-sided p <0.05. Data analysis was performed using RStudio version 4.1 [RStudio].^26^

## 3. Results

**Figure 2.**
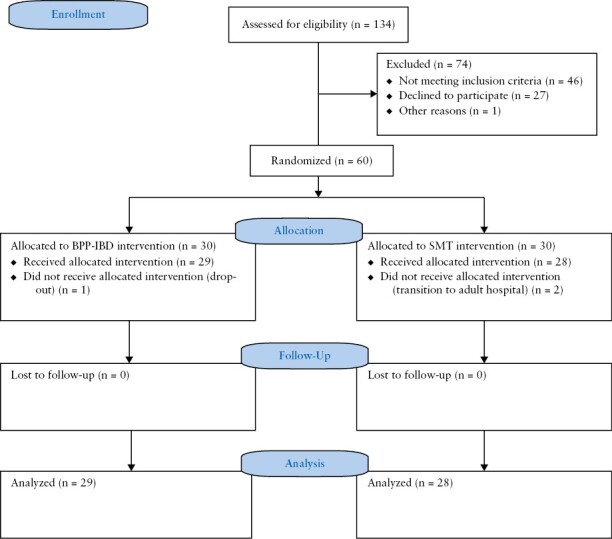
CONSORT flow chart of study design.

**Figure 3.**
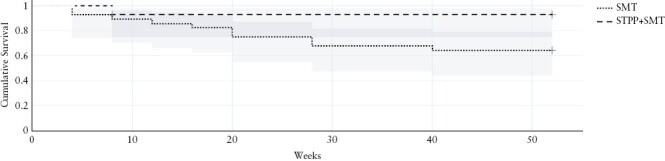
Time to disease recurrence (weeks) during the 52 weeks follow-up in SMT and STPP+SMT groups.

Participants were recruited from September 2021 to December 2021. There were no adverse effects in either treatment condition; see Figure 2 for the CONSORT flow diagram. Of the 134 patients assessed for eligibility, 60 were randomised; 74 participants were excluded from the study because they did not meet the inclusion criteria [41 were excluded due to disease relapse during the treatment period, one for history of mania/psychosis, two for steroid use, and three for smoking or other medical exclusion criteria]. Furthermore, 27 were excluded for refusal to participate in the study. All patients were in clinical remission at the time of enrolment. At 1-year follow-up, 57 [57/60] patients [95%] were considered [29 STPP + SMT, 28 SMT], three patients did not receive allocation to treatment as two moved to adult IBD centre and one dropped out before treatment start. The only demographic or clinical variables that differed between groups were age (M = 17.50 [SD = 2.98] and M = 14.69 [SD = 2.80], *p* <0.01) and duration of disease (M = 40.71 [SD = 32.29] and M = 18.93 [SD = 20.67], *p* <0.01, respectively [Table 1]. The mean age was 17 years [range 13 to 21] with an average disease duration of 2 years [y] [range 0.5 to 12 y]. No significant differences were found in other clinical and psychological baseline variables. The overall dropout rate in this study was 10% [3/30], with 3% [1/30] in the STPP + SMT and 6.6% [2/30] in the SMT arm, respectively. Primary and secondary outcomes comparisons are summarised in Table 2. Fisher’s exact test comparing the two groups on the proportion that maintained remission at 52 weeks was significant [*p* = 0.01] with 64.3% [18/28] of SMT patients and 93.1% [27/29] of STPP + SMT patients maintaining steroid-free remission at 52 weeks. The results of Fisher’s exact test did not indicate a significant improvement in STPP + SMT intervention for health care use [*p* = 0.64] and Adherence [*p* = 0.19]. Paired t test showed a significant reduction in depression and anxiety symptoms score at 52 weeks in the STPP + SMT group [Table 3]. The two-way ANOVA showed reduction of depression (F [1, 55] = 6.4, *p* = 0.014) but not anxiety (F [1, 55] = 1.15, *p* = 0.288) in STPP + SMT group. A log rank test was run to determine if there were differences in the survival distribution for the two different types of condition: STPP + SMT and SMT alone. The survival distributions [Kaplan–Meier curve] for the interventions were statistically significantly different, χ^21^ = 6.75, *p* = 0.009 [Figure 3].

**Table 1.** Sociodemographic characteristics of participants at baseline.

Baseline characteristic	SMT	STPP + SMT	*p*
	*n *= 30	*n *= 30	
Sex [male] [%]	18 [64.3]	18 [64.3]	1
Age [years] (mean [SD])	17.50 [2.98]	14.69 [2.80]	<0.01
BMI [kg/m^2^] (mean [SD])	19.15 [6.27]	16.75 [8.54]	0.23
Disease duration [months] (mean [SD])	40.71 [32.29]	18.93 [20.67]	<0.01
IBD type [%]			1
CD	10 [35.7]	11 [37.9]	
UC	18 [64.3]	18 [62.1]	
Surgical resection [%]	1 [3.6]	2 [6.9]	1
Medication			
5-ASA [%]	13 [46.4]	15 [51.7]	0.89
AZA [%]	5 [17.9]	2 [6.9]	0.39
Steroid [%]	0 [0.0]	0 [0.0]	NA
Biologics [%]	6 [21.4]	7 [24.1]	1
Previous therapeutic lines^a^ [%]	2.5 [1.2]	2.3 [1.3]	0.43
Steroid naïve^b^ [%]	7 [23.3]	9 [30]	0.61
Previous flares^c^ [%]			0.23
1	14 [46.6]	19 [63.3]	
2	2 [6.7]	0	
Steroid cycles^d^ [%]			0.58
1	9 [30]	10 [33]	
3	0	1 [3.3]	
Baseline psychological symptoms			
Depressive symptoms [PHQ-9] (mean [SD])	6.9 [4.7]	7.7 [4.4]	0.64
PHQ-9 ≥10 [%]	7 [25]	13 [44.8]	0.12
Anxiety symptoms [GAD-7] (mean [SD])	7.3 [5.8]	8 [5]	0.50
GAD-7 ≥10 [%]	8 [28.6]	8 [27.6]	0.93
Previous psychotropic medication [%]^e^	2 [7.1]	0 [0.0]	0.47

*N *= 60 [*n *= 30 for each condition]. SD = standard deviation; SMT = standard medical therapy; STPP = short-term psychodinamic psychotherapy; BMI = body mass index; PHQ-9 = patient health questionnaire; GAD-7 = generalised anxiety disorder; AZA = Azathioprine; 5-ASA = 5-aminosalicylic acid (Mesalamine).

^a,b,c,d^Refers to the 52 weeks preceding the baseline.

^e^Reflects the number and percentage of participants answering ‘yes’ to this question [including two youths treated with anxiolytic medication 4 years before baseline assessment].

**Table 2.** Comparison of primary and secondary outcomes between the two groups.

Measure	SMT	STPP + SMT	*p*
	28	29	
Remission maintenance [at 52 weeks] [%]	18 [64.3]	27 [93.1]	0.01
Health care use [%]			0.64
0	21 [75.0]	17 [58.6]	
1	3 [10.7]	5 [17.2]	
2	3 [10.7]	3 [10.3]	
3	1 [3.6]	2 [6.9]	
4+	0 [0.0]	2 [6.9]	
Adherence to medication [yes] [%]	24 [85.7]	28 [96.6]	0.19

**Table 3.** Results of paired t test of anxiety and depression symptoms according to group [baseline vs 52 weeks].

		Pre		Post		*t*	*p*
		M	SD	M	SD		
SMT	Anxiety	7.3	5.8	6.2	3.2	1.2	0.239
	Depression	6.9	4.7	6.2	3.9	1.3	0.203
STPP + SMT	Anxiety	8.0	5.0	5.6	3.0	3.4	0.002
	Depression	7.7	4.4	4.3	2.8	3.6	0.001

Bivariate analysis has shown that participants in the STPP + SMT group had 7.5 times (OR_crude_: 7.5 [95% CI 1.7–52.7]) less risk of flares in the next 52 weeks after baseline compared with SMT alone. The effect of treatment remains stable (OR_adj_: 10 [95% CI 1.3–77.5]) even including possible confounders in the multivariate model [Supplementary Material 1].

## 4. Discussion

To our knowledge, this is the first study to present changes in steroid-free remission rates after STPP in AYA with IBD. STPP in addition to SMT reduced the risk of flares approximately seven times compared with SMT alone in the following 52 weeks after baseline. Our findings are consistent with data on the efficacy of psychological and behavioural interventions on IBD disease activity, such as gut-directed hypnotherapy and cognitive behaviour therapy.^27^ Furthermore, a recent systematic review and meta-analysis of clinical trials confirmed that STPP could be considered a useful additional treatment to standard medical care for chronic health conditions, improving symptom relief and treatment outcomes.^28^ The overall high remission rates observed in this study are consistent with those reported in a recent study evaluating the efficacy of mindfulness treatment in adults with quiescent UC, which reported rates of 100% and 78% for mindfulness and controls, respectively.^29^

Proposed mechanisms for these findings include how psychotherapies may also affect the immune system. For instance, a recent systematic review and meta-analysis have found that psychological and behavioural therapies were associated with positive changes in immunity over time, including a reduction in harmful immune function that persisted for at least 6 months following treatment for participants randomly assigned to psychosocial intervention vs a control group.^30^ Interestingly, it has been suggested that dysregulation of the HPA [hypothalamic-pituitary-adrenal] axis is associated both with inflammation and depressive behaviour, underscoring the direct clinical significance of elevations in pro-inﬂammatory cytokines and suggesting a bidirectional association between depression and a pro-inflammatory state.^31,32^ In this study we observed a reduction in depression symptoms at 52 weeks of follow-up from baseline in STPP + SMT groups, probably due to the biopsychosocial dynamics of IBD, in which decreasing depression could beneficially mediate the immune system and reduce inflammation, influencing the pathway by which inflammation drives depression and depression drives inflammation.^33,34^ Recent studies found a reduction in pro-inﬂammatory cytokines blood levels after psychotherapy, demonstrating serum IL-6 [interleukin 6] and TNF-α [tumour necrosis factor-alpha] levels signiﬁcantly decreased after brief psychotherapy [eg, short-term dynamic psychotherapy, cognitive behaviour therapy].^35,36^

Contrary to our expectations, we found no significant differences in adherence and health care use between the two groups. In this study both groups showed high remission rates, reducing symptom exacerbations and limiting hospitalisations overall Furthermore, adherence was high in both groups and this may not adequately identify significant differences between groups. In addition, we speculate that the fact that intervention did not include a specific psychological intervention on adherence, such as psychoeducation, could limit adherence improvements.^37^ Dropout rate was low in this study, probably related to our individualised approach. Indeed, participants may feel more engaged and active in their care.

## 4.1. Strengths and limitations

One of the strengths of the current study is the prospective randomised design, in which the treating gastroenterologists were blinded to group assignment. Furthermore, we included patients of a wide range of ages, suggesting the efficacy of STPP from early adolescence to emerging adulthood. Baseline difference in age and disease duration between study groups may represent a limitation, although multivariate analysis excluded their influence on primary outcome. Limitations due to the small sample size must be considered. Furthermore, we were unable to include blood sample collection to investigate the effect of STPP on systemic inflammatory markers [eg, C-reactive protein, IL-6, and TNF-α] or faecal calprotectin. Another limitation was the large 95% CI of the OR, possibly related to the small sample size, which may limit the generalisation of the results. Considering the difficulty of replicating psychological therapeutic trials, a detailed protocol of our adapted STPP is currently being drafted for future researches.

## 4.2. Conclusions

Overall, AYA in the STPP + SMT group experienced improved disease outcomes 12 months after baseline, compared with standard care controls. We suggest replicating studies with larger sample sizes and multicentre designs, to provide more robust evidence on the benefits of STPP in addition to SMT for IBD paediatric patients, compared with other [psychological] interventions. Furthermore, examining the demographic, clinical, and psychological moderators, as well as the mechanisms underlying the effects of STPP, may allow for a better understanding of how STPP reduces exacerbations in AYA with IBD. Offering STPP in addition to standard care for AYA with IBD appears to be beneficial in increasing the chance of maintaining remission. Moreover, the use of an individualised approach could be a valuable support for psychological interventions with patients with IBD, to limit the dropout rate, and to enhance dissemination of psychological care of IBD. STPP could be generalised as an adjuvant treatment for relapse prevention.

Ethical approval was obtained from the Ethics Committee of Bambino Gesù Children’s Hospital, and all enrolled patients signed informed consent [protocol no. 2857]. All methods were carried out in accordance with the Declaration of Helsinki. Informed consent was obtained from all the participants and/or their legal guardians.

The datasets of this study are available from the corresponding author on reasonable request.

## Supplementary Material

jjad145_suppl_Supplementary_Table_S1

## Data Availability

The datasets of this study are availability from the corresponding author on reasonable request.

## References

[1] Rosen MJ , Dhawan A, Saeed SA. Inflammatory bowel disease in children and adolescents. JAMA Pediatr 2015;169:1053–60. doi:10.1001/jamapediatrics.2015.1982. 26414706 PMC4702263

[2] Mikocka-Walus A , Pittet V, Rossel JB, von Känel R; Swiss IBD Cohort Study Group. Symptoms of depression and anxiety are independently associated with clinical recurrence of Inflammatory Bowel Disease. Clin Gastroenterol Hepatol 2016;14:829–35.e1. doi:10.1016/j.cgh.2015.12.045. 26820402

[3] Panara AJ , Yarur AJ, Rieders B, et al. The incidence and risk factors for developing depression after being diagnosed with inflammatory bowel disease: a cohort study. Aliment Pharmacol Ther 2014;39:802–10. doi:10.1111/apt.12669. 24588323

[4] Gracie DJ , Hamlin PJ, Ford AC. The influence of the brain-gut axis in inflammatory bowel disease and possible implications for treatment. Lancet Gastroenterol Hepatol 2019;4:632–42. doi:10.1016/S2468-1253[19]30089-5. 31122802

[5] Creed F , Fernandes L, Guthrie E, et al.; North of England IBS Research Group. The cost-effectiveness of psychotherapy and paroxetine for severe irritable bowel syndrome. Gastroenterology 2003;124:303–17. doi:10.1053/gast.2003.50055. 12557136

[6] Guthrie E , Creed F, Dawson D, Tomenson B. A randomised controlled trial of psychotherapy in patients with refractory irritable bowel syndrome. Br J Psychiatry 1993;163:315–21. doi:10.1192/bjp.163.3.315. 8401959

[7] Center for Substance Abuse Treatment. Brief Interventions and Brief Therapies for Substance Abuse. Rockville MD: Substance Abuse and Mental Health Services Administration [US]; 1999. 22514840

[8] Keller W , Pritsch M, Von Wietersheim J, et al.; German Study Group on Psychosocial Intervention in Crohn's Disease. Effect of psychotherapy and relaxation on the psychosocial and somatic course of Crohn’s disease: main results of the German Prospective Multicenter Psychotherapy Treatment study on Crohn’s Disease. J Psychosom Res 2004;56:687–96. doi:10.1016/S0022-3999[03]00122-3. 15193965

[9] Kampling H , Köhler B, Germerott I, et al. An integrated psychosomatic treatment program for People with Diabetes [psy-PAD]. Dtsch Arztebl Int 2022;119:245–52. doi:10.3238/arztebl.m2022.0094. 35074044 PMC9358352

[10] Weißflog G , Brähler E, Leuteritz K, et al. Does psychodynamic short-term psychotherapy for depressed breast cancer patients also improve fatigue? Results from a randomized controlled trial. Breast Cancer Res Treat 2015;152:581–8. doi:10.1007/s10549-015-3494-0. 26163828

[11] Beutel ME , Weißflog G, Leuteritz K, et al. Efficacy of short-term psychodynamic psychotherapy [STPP] with depressed breast cancer patients: results of a randomized controlled multicenter trial. Ann Oncol 2014;25:378–84. doi:10.1093/annonc/mdt526. 24347520

[12] Levine A , Koletzko S, Turner D, et al.; European Society of Pediatric Gastroenterology, Hepatology, and Nutrition. ESPGHAN revised Porto criteria for the diagnosis of inflammatory bowel disease in children and adolescents. J Pediatr Gastroenterol Nutr 2014;58:795–806. 24231644 10.1097/MPG.0000000000000239

[13] Hyams JS , Ferry GD, Mandel FS, et al. Development and validation of a pediatric Crohn’s disease activity index. J Pediatr Gastroenterol Nutr 1991;12:439–47. 1678008

[14] Turner D , Otley AR, Mack D, et al. Development, validation, and evaluation of a pediatric ulcerative colitis activity index: a prospective multicenter study. Gastroenterology 2007;133:423–32. doi:10.1053/j.gastro.2007.05.029. 17681163

[15] De Sousa JFM , Paghdar S, Khan TM, Patel NP, Chandrasekaran S, Tsouklidis N. Stress and inflammatory bowel disease: clear mind, happy colon. Cureus 2022;14:e25006. doi:10.7759/cureus.25006. 35582022 PMC9107617

[16] Mawdsley JE , Rampton DS. The role of psychological stress in inflammatory bowel disease. Neuroimmunomodulation 2006;13:327–36. doi:10.1159/000104861. 17709955

[17] Sun Y , Li L, Xie R, Wang B, Jiang K, Cao H. Stress triggers flare of inflammatory bowel disease in children and adults. Front Pediatr 2019;7:432. doi:10.3389/fped.2019.00432. 31709203 PMC6821654

[18] Bernstein CN. Psychological stress and depression: risk factors for IBD? Dig Dis 2016;34:58–63. doi:10.1159/000442929. 26983009

[19] Malan DH. The Frontier of Brief Psychotherapy. New York, NY: Plenum; 1976.

[20] Fosha D. The dyadic regulation of affect. J Clin Psychol 2001;57:227–42. 11180149 10.1002/1097-4679(200102)57:2<227::aid-jclp8>3.0.co;2-1

[21] Renna ME. A review and novel theoretical model of how negative emotions influence inflammation: the critical role of emotion regulation. Brain Behav Immun Health 2021;18:100397. doi:10.1016/j.bbih.2021.100397. 34927103 PMC8649080

[22] Spitzer RL , Kroenke K, Williams JB, Löwe B. A brief measure for assessing generalized anxiety disorder: the GAD-7. Arch Intern Med 2006;166:1092–7. 16717171 10.1001/archinte.166.10.1092

[23] Kroenke K , Spitzer RL, Williams JB. The PHQ-9: validity of a brief depression severity measure. J Gen Intern Med 2001;16:606–13. 11556941 10.1046/j.1525-1497.2001.016009606.x PMC1495268

[24] Gurram B , Patel AS. Recent advances in understanding and managing pediatric inflammatory bowel disease. F1000Res 2019;8:F1000. Faculty Rev-2097. doi:10.12688/f1000research.19609.1. PMC6913196 31885858

[25] Keefer L , Taft TH, Kiebles JL, Martinovich Z, Barrett TA, Palsson OS. Gut-directed hypnotherapy significantly augments clinical remission in quiescent ulcerative colitis. Aliment Pharmacol Ther 2013;38:761–71. doi:10.1111/apt.12449. 23957526 PMC4271841

[26] RStudio Team. RStudio: Integrated Development for R. Boston, MA: RStudio; 2020.

[27] Ballou S , Keefer L. Psychological interventions for irritable bowel syndrome and Inflammatory Bowel Diseases. Clin Transl Gastroenterol 2017;8:e214. doi:10.1038/ctg.2016.69. 28102860 PMC5288603

[28] Abbass A , Town J, Holmes H, et al. Short-term psychodynamic psychotherapy for functional somatic disorders: a meta-analysis of randomized controlled trials [Erratum. Psychother Psychosom. 2020;89(6):408. doi:10.1159/000508894. Psychother Psychosom 2020;89:363–70. doi:10.1159/000508894. 32428905

[29] Jedel S , Beck T, Swanson G, et al. Mindfulness intervention decreases frequency and severity of flares in inactive ulcerative colitis patients: results of a phase II, randomized, placebo-controlled trial. Inflamm Bowel Dis 2022;28:1872–92. doi:10.1093/ibd/izac036. 35661212 PMC9713500

[30] Shields GS , Spahr CM, Slavich GM. Psychosocial interventions and immune system function: a systematic review and meta-analysis of randomized clinical trials. JAMA Psychiatry 2020;77:1031–43. doi:10.1001/jamapsychiatry.2020.0431. 32492090 PMC7272116

[31] Moreira FP , Cardoso Tde A, Mondin TC, et al. The effect of proinflammatory cytokines in Cognitive Behavioral Therapy. J Neuroimmunol 2015;285:143–6. doi:10.1016/j.jneuroim.2015.06.004. 26198931

[32] Del Grande da Silva G , Wiener CD, Barbosa LP, et al. Pro-inflammatory cytokines and psychotherapy in depression: results from a randomized clinical trial. J Psychiatr Res 2016;75:57–64. doi:10.1016/j.jpsychires.2016.01.008. 26802811

[33] Gracie DJ , Guthrie EA, Hamlin PJ, Ford AC. Bi-directionality of brain-gut interactions in patients with inflammatory bowel disease. Gastroenterology 2018;154:1635–46.e3. doi:10.1053/j.gastro.2018.01.027. 29366841

[34] Keefer L , Kane SV. Considering the bidirectional pathways between depression and IBD: recommendations for comprehensive IBD care. Gastroenterol Hepatol [N Y] 2017;13:164–9. PMC5439135 28539843

[35] Colasanto M , Madigan S, Korczak DJ. Depression and inflammation among children and adolescents: a meta-analysis. J Affect Disord 2020;277:940–8. doi:10.1016/j.jad.2020.09.025. 33065836

[36] Dantzer R , O’Connor JC, Freund GG, Johnson RW, Kelley KW. From inflammation to sickness and depression: when the immune system subjugates the brain. Nat Rev Neurosci 2008;9:46–56. doi:10.1038/nrn2297. 18073775 PMC2919277

[37] Nieuwlaat R , Wilczynski N, Navarro T, et al. Interventions for enhancing medication adherence. Cochrane Database Syst Rev 2014;2014:CD000011. doi:10.1002/14651858.CD000011.pub4. 25412402 PMC7263418

