# Supplementary material for: Short-term Psychodynamic Psychotherapy in Addition to Standard Medical Therapy Increases Clinical Remission in Adolescents and Young Adults with Inflammatory Bowel Disease: a Randomised Controlled Trial
Source: J Crohns Colitis. 2023 Aug 24;18(2):256–63. doi: 10.1093/ecco-jcc/jjad145 (PMC10896630; doi:10.1093/ecco-jcc/jjad145)
Supplement: jjad145_suppl_Supplementary_Table_S1 [file jjad145_suppl_supplementary_table_s1.docx]

S1. Multivariate analysis of clinical and psychological variables on disease flares in the 52 weeks after baseline.

| Predictors | *OR* | 95% CI | | *p* |
| --- | --- | --- | --- | --- |
|  |  | *LL* | *UL* |  |
|  |  |  |  |  |
| Intervention (STPP+SMT) | 10.0 | 1.3 | 77.5 | .03 |
| Age (years) | 1.1 | .8 | 1.4 | .45 |
| Disease duration (months) | 1.0 | .9 | 1.0 | .83 |
| Sex (male) | 1.5 | .3 | 7.7 | .61 |
| Disease type (CD) | .6 | .1 | 2.7 | .53 |
| Biologics use (yes) | .3 | .1 | 2.0 | .22 |
| Baseline Anxiety symptoms (GAD-7≥10) | .8 | .1 | 9.1 | .86 |
| Baseline Depressive symptoms (PHQ-9≥10) | 1.6 | .0 | 50.9 | .68 |
|  |  |  |  |  |

*Note.* CI = confidence interval; *LL* = lower limit; *UL* = upper limit.
